# A longitudinal study on associations of moderate-to-vigorous physical activity with plasma monounsaturated fatty acids in pregnancy

**DOI:** 10.3389/fnut.2022.983418

**Published:** 2022-10-24

**Authors:** Tong Xia, Liwei Chen, Zhe Fei, Xinyue Liu, Jin Dai, Stefanie N. Hinkle, Yeyi Zhu, Jing Wu, Natalie L. Weir, Michael Y. Tsai, Cuilin Zhang

**Affiliations:** ^1^Department of Epidemiology, Fielding School of Public Health, University of California, Los Angeles, Los Angeles, CA, United States; ^2^Department of Biostatistics, Fielding School of Public Health, University of California, Los Angeles, Los Angeles, CA, United States; ^3^Department of Biostatistics, Epidemiology and Informatics, Perelman School of Medicine, University of Pennsylvania, Philadelphia, PA, United States; ^4^Division of Research, Kaiser Permanente Northern California, Oakland, CA, United States; ^5^Glotech, Inc., Rockville, MD, United States; ^6^Department of Laboratory Medicine & Pathology, University of Minnesota, Minneapolis, MN, United States; ^7^Global Center for Asian Women’s Health, Bia-Echo Asia Centre for Reproductive Longevity & Equality, Yong Loo Lin School of Medicine, National University of Singapore, Singapore, Singapore; ^8^Department of Obstetrics and Gynecology, Yong Loo Lin School of Medicine, National University of Singapore, Singapore, Singapore

**Keywords:** fatty acids, monounsaturated fatty acids, physical activity, moderate-to-vigorous physical activity, pregnant women

## Abstract

**Background:**

Physical activity (PA) during pregnancy influences women and offspring’s health *via* fatty acids metabolism. However, studies on associations of PA with plasma monounsaturated fatty acids (MUFAs) across pregnancy are sparse. Thus, our study aimed to examine associations of PA with individual plasma phospholipid MUFAs throughout pregnancy in a prospective and longitudinal study in the United States (US).

**Materials and methods:**

The study included 318 pregnant women from the *Eunice Kennedy Shriver* National Institute of Child Health and Human Development Fetal Growth Studies-Singletons cohort. PA was measured four times: PA reported at 10–14 gestational weeks (GWs) representing PA in the past year, and at 15–26 GWs, 23–31 GWs, and 33–39 GWs representing PA since the last visit. Plasma phospholipid MUFAs were measured at the same four visits as the measurement of PA. Associations between moderate-to-vigorous PA (MVPA) and the total MUFAs and seven individual plasma phospholipid MUFAs (i.e., palmitoleic acid, 18:1n6-9 *trans*, 18:1n6c, *cis-*vaccenic acid, oleic acid, eicosenoic acid, and nervonic acid) were assessed at each visit using multivariable linear regression models adjusting for confounders.

**Results:**

MVPA (hours/week) reported at 15–26 GWs representing MVPA since the last visit was positively associated with total MUFAs (% of total fatty acids) [adjusted β*10^2^ (standard error (SE)*10^2^) = 10.41 (3.19), *P* = 0.001] at 15–26 GWs. For individual MUFAs, MVPA reported at 15–26 GWs representing MVPA since the last visit was positively associated with oleic acid [adjusted β*10^2^ (SE*10^2^) = 8.56 (2.65), *P* = 0.001] and eicosenoic acid [adjusted β*10^2^ (SE*10^2^) = 0.55 (0.20), *P* = 0.01] at 15–26 GWs. MVPA reported at 23–31 GWs representing MVPA since the last visit was positively associated with palmitoleic acid [adjusted β*10^2^ (SE*10^2^) = 2.24 (0.64), *P* = 0.001] at 23–31 GWs. MVPA reported at 10–14 GWs and 33–39 GWs was not associated with total or individual MUFAs.

**Conclusion:**

We found novel positive associations of MVPA with individual MUFAs, such as oleic acid, eicosenoic acid, and palmitoleic acid, during middle-to-late pregnancy. These findings suggest that MVPA represents a potentially modifiable factor for plasma individual MUFA levels during pregnancy.

## Introduction

Monounsaturated fatty acids (MUFAs) have multiple biological functions, including the formation of biological membranes and triglyceride (TG) synthesis (1, 2). Evidence showed that maternal plasma levels of MUFAs during pregnancy were critical for maternal health and fetal growth (3–5). Further, individual MUFAs might have distinct health effects. For example, plasma oleic acid was associated with a decreased risk of diabetes and stroke (6, 7), whereas nervonic acid was associated with an increased risk of congestive heart failure, depressive disorder, and all-cause mortality (8–10). Such observations were also reported for pregnant women even though data among pregnant women were scant (4, 11). For instance, regarding individual MUFAs, only maternal plasma oleic acid was inversely associated with gestational diabetes mellitus (GDM) (4). Maternal plasma oleic acid and palmitoleic acid were also inversely associated with biomarkers of inflammation, such as interleukin (IL)-8, IL-6, and tumor necrosis factor-alpha (11).

These recent findings point to the importance of gaining a better understanding of modifiable determinants of circulating individual MUFA levels among different populations, including pregnant women. Although dietary intake is the major source of circulating MUFAs, other factors play important roles through endogenous synthesis and metabolism (2, 12, 13).

Physical activity (PA) is an important determinant for cardiovascular health (14, 15) and lipid metabolism in humans (14, 15). During pregnancy, leisure time PA is also beneficial for pregnant women and fetuses (e.g., lower risk of excessive gestational weight gain, GDM, preeclampsia, preterm birth, and low birthweight) (16–18). Emerging evidence suggested that PA might also influence circulating individual MUFA levels in non-pregnant populations (19–24). In crossover studies, acute exercise at moderate intensity resulted in higher serum palmitoleic acid, oleic acid, and eicosenoic acid levels compared to rest among healthy individuals (20, 21). Long-term intensive exercise changed plasma individual phospholipid MUFA profiles in professional football players (24). However, no studies have examined the relationship between PA and individual circulating MUFAs during pregnancy. To address this knowledge gap, we aimed to comprehensively examine the associations of PA with individual MUFAs among pregnant women at multiple time-points throughout pregnancy. We hypothesized that individual MUFAs may have distinct associations with PA and the associations may vary over time during pregnancy.

## Materials and methods

### Study design and population

Study participants were from the prospective *Eunice Kennedy Shriver* National Institute of Child Health and Human Development (NICHD) Fetal Growth Studies-Singletons cohort, which was conducted between 2009 and 2013. The cohort recruited 2,802 pregnant women between the ages of 18 and 40 who were generally healthy and without pre-existing diseases (25). Pregnant women were enrolled at 8–13 gestational weeks (GWs) at 12 clinical centers across the United States (US) and were followed until delivery (92.3% of women having data at delivery) (25). We leveraged data within a subsample (*n* = 321) of the cohort from a GDM nested case-control study which measured PA and MUFAs. One GDM case was matched with two non-GDM controls on age (±2 years), race/ethnicity, and GWs (±2 weeks) at blood collection. After excluding women without MUFAs measured at baseline (*n* = 3), the analytical population included 318 women who had both PA and individual MUFAs measured at baseline (Supplementary Figure 1). Among these, no women had a spontaneous abortion, and only a very small percentage of women (4.4%, *n* = 14) had preterm birth (i.e., < 37 GW). As GDM women were overrepresented in the study sample, we applied sampling weights (i.e., inverse probability of each subject selected from the full cohort) to the primary analyses so the results of this study can be generalized to the full cohort (26). More details on study design and participant characteristics have been previously published (25). The institutional review boards of NICHD and all participating clinical sites approved the study. All participants provided written informed consent.

### Assessment of physical activity

We assessed PA among pregnant women longitudinally four times during the pregnancy *via* the self-administrated Pregnancy PA Questionnaire (PPAQ) (27). At 10–14 GWs, women reported their PA in the previous year (i.e., habitual PA in preconception and first trimester). At 15–26 GWs, 23–31 GWs, and 33–39 GWs, women reported their PA since the prior visit (25). In the current investigation, we classified moderate [3.0–6.0 metabolic equivalents task (MET), e.g., walking slowly or quickly for fun/exercise, swimming, dancing] and vigorous (> 6.0 MET, e.g., walking up hills for fun/exercise, jogging) PA from sports/exercise activities (28, 29). We calculated the time spent on moderate-to-vigorous PA (MVPA) and treated those as continuous variables (hours/week). We also calculated the time spent on sedentary behavior (hours/week).

### Assessment of plasma phospholipid monounsaturated fatty acids

We measured plasma phospholipid fatty acids from blood specimens collected among participants longitudinally during pregnancy during the same four study visits as the assessment of PA (25). Due to the consideration of reducing participants’ burden and maintaining a high follow-up rate, only blood samples collected at 15–26 GWs were fasting (i.e., overnight fast of 8–14 h). We extracted and measured plasma phospholipid MUFAs using a Hewlett Packard 5,890 gas chromatography system with flame ionization detection (GC-FID) which was a method previously described (30). Briefly, we extracted total lipids from plasma using a chloroform:methanol extraction, and separated phospholipids fraction using thin-layer chromatography. The phospholipids were isolated and converted to fatty acid methyl esters. The fatty acid methyl esters were further separated by GC-FID. We identified fatty acids using mixtures of known fatty acid methyl esters from Nu-Chek Prep (Elysian Township, MN, USA). Levels of individual phospholipid MUFAs were expressed as a percentage (%) of the total phospholipid FAs. We identified seven individual MUFAs, including *trans*-MUFA: 18:1n6-9 *trans* (sum of 18:1n-6 *trans*, 18:1n-7 *trans*, 18:1n-8 *trans*, and 18:1n-9 *trans*), and six *cis*-MUFAs: 16:1n7c (Palmitoleic acid), 18:1n6c, 18:1n7c (*cis*-Vaccenic acid), 18:1n9c (Oleic acid), 20:1n9 (Eicosenoic acid), and 24:1n9 (Nervonic acid). An in-house pooled plasma control was assessed with each batch throughout the project and the coefficient of variation (CV) was 6.4% for 18:1n6-9 *trans*, 6.1 for 16:1n7c, 11.8% for 18:1n6c, 6.0% for 18:1n7c, 4.4% for 18:1n9c, 27.9% for 20:1n9, and 33.3% for 24:1n9.

### Covariates

We collected sociodemographic, anthropometric, reproductive, and lifestyle factors from detailed questionnaires at baseline (i.e., 8–13 GWs). We calculated pre-pregnancy body mass index (BMI) by self-reported pre-pregnancy weight and measured height at enrollment. At baseline, we assessed habitual dietary intakes in the prior 3 months using a validated Food Frequency Questionnaire (FFQ) (31, 32). At follow-up visits (i.e., 16–22 GWs, 24–29 GWs, and 34–37 GWs), we assessed dietary intake from the past 24 h using the automated self-administered 24-h dietary recall (ASA24) based on the validated US Department of Agriculture (USDA) Automated Multiple-Pass Method (33). We calculated the Alternative Health Eating Index (AHEI) without alcohol from a validated method as an index of overall dietary quality during pregnancy (34).

### Statistical analysis

As mentioned in the above section, we applied sampling weights using the inverse probability method so the results of this study can be generalizable to the full NICHD Fetal Growth Studies-Singletons cohort (26). We conducted all analyses using SAS version 9.4 (SAS Institute, Cary, NC, USA) and considered the statistically significant level after controlling for multiple testing (i.e., Bonferroni-corrected α level < 0.05) (35).

We presented baseline characteristics and described the distribution of MVPA, sedentary behavior, and levels of individual MUFAs throughout pregnancy. For descriptive analyses, we reported the weighted percentage and actual frequency [% (N)] for categorical variables and weighted mean and standard errors (SE) for continuous variables.

For primary analyses, we examined the associations of MVPA as a continuous variable (hours/week) with plasma total and individual phospholipid MUFAs (% of total FAs) using linear regression models with and without adjustment for confounders, including age (years), race/ethnicity (Non-Hispanic White, Non-Hispanic Black, Hispanic, and Asian/Pacific Islander), education (high school or less; associates degree; bachelor’s or higher degree), marital status (married/living with a partner or not), parity (nulliparous or multiparous), pre-pregnancy BMI (kg/m^2^), AHEI score, and sedentary behavior (hours/week). We chose covariates that made the estimate change more than 10% as real confounders (36, 37). For association analyses at each visit, we excluded those participants without data on PA or MUFAs. At 15–26 GWs and 23–31 GWs, less than 5% of participants were missing data on PA (i.e., MVPA and sedentary behavior) and MUFAs. At 33–39 GWs, 12.6 and 7.5% of participants were missing data on PA and MUFAs, respectively (Supplementary Table 1). Meanwhile, since dietary intakes were only available among 197, 210, 146, and 129 women at 8–13 GWs, 16–22 GWs, 24–29 GWs, and 34–37 GWs, respectively (Supplementary Table 1). Due to the dietary assessment tools deployed part way through the study, we used mean imputation for missing values when controlling for dietary factors. In the sensitivity analyses, we applied multiple imputations for the missing data. Additionally, based on primary analyses, we further controlled for the dietary intake of total MUFAs or individual FAs, and total energy intake. Furthermore, we conducted primary analyses only on controls (i.e., women without GDM) and did not apply sampling weights. Besides controlling for the sedentary behavior in the above regression models, we also examined the associations of MVPA with MUFAs stratified by the median value of sedentary behavior (i.e., high sedentary behavior: ≥ 42 h of sedentary behavior per week; low sedentary behavior: < 42 h of sedentary behavior per week). We tested the interaction of MVPA and sedentary behavior with MUFAs using the multiplicative method. The *P* for interaction was calculated using the Wald Chi-squared test. The study was reported according to the Strengthening the Reporting of Observational Studies in Epidemiology (STROBE) statement.

## Results

### Baseline characteristics of study participants

The weighted mean (SE) age of women at baseline was 28.2 (0.3) years and the mean (SE) of pre-pregnancy BMI was 25.7 (0.3) kg/m^2^. Participants were from four racial/ethnic groups, with 31.0% Non-Hispanic White, 27.2% Hispanic, 23.3% Non-Hispanic Black, and 18.5% as Asian/Pacific Islander. The mean (SE) of the AHEI score was 44.1 (0.7) (Table 1).

**TABLE 1:** Baseline characteristics of the analytical population from the NICHD Fetal Growth Studies-Singleton Cohort.^1^

Characteristics	*N* = 318
	
Age, years	28.2 (0.3)
Race/ethnicity, % (*N*)	
Non-Hispanic White	31.0 (75)
Non-Hispanic Black	23.3 (45)
Hispanic	27.2 (122)
Asian/Pacific Islander	18.5 (76)
Pre-pregnancy BMI, kg/m^2^	25.7 (0.3)
Education, % (*N*)	
High school or less	45.5 (146)
Associates	14.7 (50)
Bachelor’s or higher	39.8 (122)
Married/living with a partner, % (*N*)	72.9 (257)
Nulliparous, % (*N*)	51.1 (142)
Dietary MUFAs^2^	29.6 (1.1)
AHEI score^2^	44.1 (0.7)

^1^Data were presented as weighted percentage and actual frequency, % (N) for categorical variables, and weighted mean (SE) for continuous variables. Sampling weights were applied to all analyses to represent the full NICHD Fetal Growth Studies-Singletons population. ^2^Dietary intakes were collected among 197 women who completed the Food Frequency Questionnaires at baseline. Seven women with implausible extreme total energy intake (i.e., < 600 or > 6,000 kcal/day) were additionally excluded. AHEI, Alternative Health Eating Index; BMI, body mass index; MUFAs, monounsaturated fatty acids; NICHD, National Institute of Child Health and Human Development; SE, standard error.

### Changes in moderate-to-vigorous PA and monounsaturated fatty acids throughout pregnancy

The mean (SE) of MVPA reported at baseline, 10–14 GWs (representing MVPA from the previous year), was 2.92 (0.15) hours/week. Time spent on MVPA decreased slightly during pregnancy (Supplementary Table 2). Among the seven individual MUFAs, oleic acid (18:1n9c) was the most abundant form and accounted for 7.31% of the total plasma phospholipid FAs level at 10–14 GWs, followed by *cis*-vaccenic acid (18:1n7c) at 1.40% and nervonic acid (24:1n9) at 1.10%, whereas other individual MUFAs contributed less than 1.00%, with the lowest concentration for eicosenoic acid at 0.15% (Supplementary Table 3). Overall, changes in MUFA levels across pregnancy were subtle. Oleic acid showed an increasing trend; *trans*-MUFA (sum of 18:1n6 *trans*, 18:1n7 *trans*, 18:1n8 *trans*, and 18:1n9 *trans*), 18:1n6c, *cis*-vaccenic acid, and eicosenoic acid showed a decreasing trend (all *P*-values for trend ≤ 0.007) (Supplementary Figure 2).

### Prospective associations of habitual moderate-to-vigorous PA with plasma phospholipid monounsaturated fatty acids

The unadjusted and adjusted associations of MVPA with total and individual MUFAs are shown in Table 2. In unadjusted models, MVPA (hours/week) reported at 10–14 GWs representing past year MVPA (i.e., preconception and first trimester) was positively associated with total MUFAs (% of total FAs) and oleic acid (% of total FAs) at 10–14 GWs. MVPA reported at 15–26 GWs representing MVPA since the last visit was positively associated with total MUFAs, oleic acid, and eicosenoic acid at 15–26 GWs. MVPA reported at 23–31 GWs representing MVPA since the last visit was positively associated with palmitoleic acid and 18:1n6c at 23–31 GWs. MVPA reported at 33–39 GWs representing MVPA since the last visit was positively associated with total MUFAs and oleic acid at 33–39 GWs. Adjusting for age, race/ethnicity, education, marital status, parity, pre-pregnancy BMI, AHEI score, and sedentary behavior, MVPA (hours/week) reported at 10–14 GWs representing past year MVPA and at 33–39 GWs representing MVPA since the last visit was no longer associated with total MUFAs (% of total FAs) or any individual MUFAs (% of total FAs). MVPA reported at 15–26 GWs representing MVPA since the last visit remained positively associated with total MUFAs [β*10^2^ (SE*10^2^) = 10.41 (3.19), *P* = 0.001] at 15–26 GWs. Additionally, for the individual MUFAs, MVPA reported at 15–26 GWs representing MVPA since the last visit was positively associated with oleic acid [β*10^2^ (SE*10^2^) = 8.56 (2.65), *P* = 0.001] and eicosenoic acid [β*10^2^ (SE*10^2^) = 0.55 (0.20), *P* = 0.01] at 15–26 GWs; MVPA reported at 23–31 GWs representing MVPA since the last visit was positively associated with palmitoleic acid [β*10^2^ (SE*10^2^) = 2.24 (0.65), *P* = 0.001] at 23–31 GWs. In sensitivity analyses, the results remained unchanged after using multiple imputations for the missing data (Supplementary Table 4) or further controlling for dietary intake of total MUFAs or individual FAs (Supplementary Table 5) or total energy intake (Supplementary Table 6). When the analyses were conducted among women without GDM, the directions of associations between MVPA and MUFAs were unchanged but became statistically insignificant (Supplementary Table 7).

**TABLE 2:** Associations of moderate-to-vigorous physical activity (MVPA) with plasma monounsaturated fatty acids (MUFAs).^1^

	10–14 gestational weeks *N* = 318						15–26 gestational weeks *N* = 308					
		
	Model 1			Model 2			Model 1			Model 2		
				
	β *10^2^	(SE*10^2^)	*P*	β *10^2^	(SE*10^2^)	*P*	β *10^2^	(SE*10^2^)	*P*	β *10^2^	(SE*10^2^)	*P*
**MVPA (hours/week)**												
Total MUFAs	6.81	(2.19)	0.002*	5.05	(2.35)	0.03	15.13	(3.21)	< 0.001*	10.41	(3.19)	0.001*
16:1n7c (Palmitoleic acid)	0.94	(0.42)	0.02	0.72	(0.44)	0.10	1.07	(0.45)	0.02	0.50	(0.43)	0.25
*Trans* MUFA^2^	0.58	(0.45)	0.20	0.55	(0.47)	0.24	0.27	(0.68)	0.69	0.36	(0.70)	0.61
18:1n6c	0.36	(0.16)	0.03	0.27	(0.17)	0.12	0.36	(0.23)	0.11	0.26	(0.24)	0.28
18:1n7c (*cis*-Vaccenic acid)	0.01	(0.41)	0.98	−0.30	(0.44)	0.50	1.26	(0.54)	0.02	0.62	(0.55)	0.26
18:1n9c (Oleic acid)	4.92	(1.76)	0.01*	3.55	(1.92)	0.06	12.26	(2.68)	< 0.001*	8.56	(2.65)	0.001*
20:1n9 (Eicosenoic acid)	0.02	(0.14)	0.86	−0.15	(0.15)	0.31	0.72	(0.18)	< 0.001*	0.55	(0.20)	0.01*
24:1n9 (Nervonic acid)	−0.03	(1.03)	0.98	0.42	(1.13)	0.71	−0.82	(0.50)	0.10	−0.44	(0.52)	0.40

	**23–31 gestational weeks *N* = 204**						**33–39 gestational weeks *N* = 187**					
		
	**Model 1**			**Model 2**			**Model 1**			**Model 2**		
				
	**β** *10^2^	(SE*10^2^)	***P***	**β** *10^2^	(SE*10^2^)	***P***	**β** *10^2^	(SE*10^2^)	***P***	**β** *10^2^	(SE*10^2^)	***P***

**MVPA (hours/week)**												
Total MUFAs	7.16	(3.46)	0.04	6.71	(3.55)	0.06	16.17	(3.92)	< 0.001*	6.98	(4.40)	0.11
16:1n7c (Palmitoleic acid)	2.31	(0.66)	< 0.001*	2.24	(0.65)	0.001*	0.96	(0.83)	0.25	−0.20	(0.87)	0.82
*Trans* MUFA^2^	0.51	(0.66)	0.45	−0.22	(0.65)	0.73	0.72	(0.74)	0.33	−0.19	(0.78)	0.81
18:1n6c	0.93	(0.33)	0.01*	0.80	(0.34)	0.02	0.72	(0.30)	0.02	0.16	(0.32)	0.61
18:1n7c (*cis*-Vaccenic acid)	−1.08	(0.56)	0.05	−0.47	(0.60)	0.44	−0.14	(0.54)	0.79	0.31	(0.60)	0.60
18:1n9c (Oleic acid)	2.94	(2.98)	0.32	3.31	(2.78)	0.23	14.56	(3.34)	< 0.001*	5.71	(3.55)	0.11
20:1n9 (Eicosenoic acid)	0.39	(0.15)	0.01	0.32	(0.16)	0.05	0.30	(0.17)	0.07	0.19	(0.19)	0.31
24:1n9 (Nervonic acid)	1.16	(1.39)	0.40	0.73	(1.42)	0.61	−0.96	(1.75)	0.59	0.99	(1.88)	0.60

^1^Model 1: unadjusted models. Model 2: multivariable models adjusted for age (years), race, education, marriage status, nulliparous, pre-pregnancy BMI (kg/m^2^), AHEI score, and sedentary behavior (hour/week). AHEI score used mean imputation for missing values. Analytical sample size for each time point was 318, 308, 204, and 187 at 10–14 GWs, 15–26 GWs, 23–31 GWs, and 33–39 GWs, respectively. Sampling weights were applied to all analyses to represent the full NICHD Fetal Growth Studies-Singletons population. *P-value is significant after Bonferroni correction. Previous year’s habitual PA was measured at baseline and PA since the last visit was measured in following visits. Individual MUFA was measured as % of total fatty acids. ^2^Trans-MUFA: 18:1n6-9 trans (sum of 18:1n-6 *trans*, 18:1n-7 *trans*, 18:1n-8 *trans*, and 18:1n-9 *trans*). AHEI, Alternative Health Eating Index; BMI, body mass index; MUFAs, monounsaturated fatty acids; MVPA, moderate-to-vigorous physical activity; NICHD, National Institute of Child Health and Human Development; PA, physical activity; SE, standard error.

**FIGURE 1:**
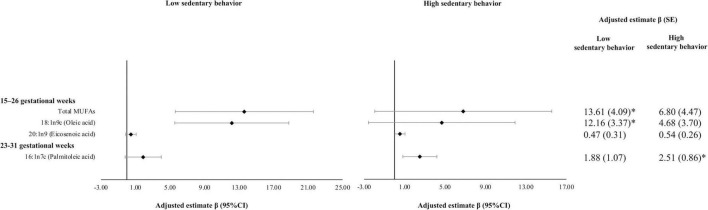
Associations of moderate-to-vigorous physical activity (MVPA) with plasma monounsaturated fatty acids (MUFAs) by sedentary behavior. Multivariable models adjusted for age (years), race, education, marriage status, nulliparous, pre-pregnancy BMI (kg/m^2^), and AHEI score. AHEI score used mean imputation for missing values. Sampling weights were applied to all analyses to represent the full NICHD Fetal Growth Studies–Singletons population. Units of continuous MVPA used hours per week. β*10^2^ (SE*10^2^) were reported. Women were classified into high sedentary behavior group if they had ≥ 42 h of sedentary behavior per week. *P-value is significant after Bonferroni correction. PA since the prior visit was measured at the two visits. Individual MUFAs were expressed as % of total fatty acids. AHEI, Alternative Health Eating Index; BMI, body mass index; MUFAs, monounsaturated fatty acids; MVPA, moderate-to-vigorous physical activity; NICHD, National Institute of Child Health and Human Development; PA, physical activity; SE, standard error.

Associations of MVPA with MUFAs did not differ significantly by sedentary behaviors although they appeared stronger among women with low sedentary behavior. The positive association between MVPA (hours/week) reported at 15–26 GWs representing MVPA since the last visit and oleic acid (% of total FAs) at 15–26 GWs was statistically significant among women with low sedentary behavior; whereas this association was relatively weaker and statistically insignificant among women with high sedentary behavior [β*10^2^ (SE*10^2^): 12.16 (3.37) vs. 4.68 (3.70)]. The test for interaction was not statistically significant (*P* for interaction = 0.36) (Figure 1).

## Discussion

In this prospective study with the longitudinal measurement of PA and plasma phospholipid MUFAs throughout pregnancy, we found that MVPA was positively associated with total MUFAs in the second trimester even after adjustment for important confounders, including age, race/ethnicity, education, marital status, parity, pre-pregnancy BMI, AHEI score, and sedentary behavior. The associations were primarily due to the positive associations of MVPA with individual MUFAs, oleic acid, eicosenoic acid, and palmitoleic acid. Our findings are significant as the metabolism of MUFAs during pregnancy is crucial for both pregnant women and fetuses (3, 5). Specifically, high levels of lipogenesis and fat deposition occur during the first two trimesters to prepare for fetal transfer during the third trimester. During the third trimester, there are high levels of lipolysis. Maternal FAs are transferred to the fetus through the placenta and are important for fetal growth and development (3, 5).

Up to now, no studies have examined associations of PA with circulating individual MUFA levels among pregnant women. A Spanish study reported no significant difference in serum total MUFAs between 35 active and 404 inactive pregnant women (38). The major limitations of this Spanish study included the small number of women in the active group (*n* = 35), PA only measured at baseline in the first trimester, and without controlling for potential confounders (38). To the best of our knowledge, the present study is the first to find positive associations of MVPA with plasma oleic acid, eicosenoic acid, and palmitoleic acid in pregnant women after controlling for major confounders, including dietary factors, suggesting that MVPA might represent a new modifiable lifestyle factor for circulating individual MUFA levels.

Our findings of the positive associations of MVPA with oleic acid, eicosenoic acid, and palmitoleic acid in pregnant women are novel and consistent with results from several small studies among non-pregnant populations (20–24). In two randomized crossover studies among healthy individuals in Austria (*n* = 13) and Norway (*n* = 8), serum and plasma free oleic acid, eicosenoic acid, and palmitoleic acid levels were higher after exercise with moderate intensity varying from 60 to 90 min than in resting states (20, 21). In addition, two studies found higher levels of plasma and serum free oleic acid or palmitoleic acid immediately after exercise among handball players (*n* = 19) or healthy middle-aged individuals (*n* = 22) with pre- and post-intervention comparisons (22, 23). Long-term PA may also influence plasma FA profiles. A study in the US found higher plasma phospholipid palmitoleic acid 2 weeks after the competitive season among football players (*n* = 24) than sedentary, healthy individuals who did not receive any training (*n* = 16) (24). Although these studies were not designed with randomized, parallel control groups and have small sample sizes, the consistent findings among different populations are reassuring.

The biological mechanisms underlying positive associations between MVPA and plasma phospholipid MUFAs among pregnant women are complex because plasma individual MUFAs could derive from both exogenous sources (i.e., dietary intake, major sources) and endogenous synthesis as shown in Supplementary Figure 3 (2, 12, 13). Δ9-desaturase in the liver is the rate-limiting step for the endogenous synthesis of palmitoleic acid from saturated FAs (SFAs) (2, 13, 39, 40). Levels of phospholipid MUFAs are also influenced by the downstream chain elongation activity. Palmitoleic acid could be elongated to *cis*-vaccenic acid by the elongation of very long chain fatty acids protein-5, 6 (ELOVL-5, 6) (13, 41, 42).

We found that MVPA was positively associated with Δ9-desaturase [estimated as palmitoleic acid/palmitic acid (2, 13, 39)] and inversely associated with ELOVL-5, 6 [estimated as *cis*-vaccenic acid/palmitoleic acid (13, 41, 42)] at 23–31 GWs (Supplementary Table 8). Previous studies also suggested that PA was associated with increased levels of peroxisome proliferator-activated receptor gamma (PPARγ) (43), and the PPARγ agonists were associated with increased mRNA levels of Δ9-desaturase enzyme stearoyl-CoA desaturase 1 (SCD1) (44, 45). These results supported the possibility that MVPA may increase palmitoleic acid by increasing Δ9-desaturase and decreasing ELVOL-5, 6 activity at 23–31 GWs. Nevertheless, these findings should be interpreted cautiously until replicated with direct measures of enzyme activities.

Besides delivered from liver synthesis, plasma phospholipid FAs compositions are also influenced by free FAs (FFAs) delivered from adipose tissue (46–49). Circulating levels of FFAs are influenced by muscle uptake and adipose tissue lipolysis (46, 50). It is likely that habitual MVPA may increase plasma free MUFA levels through the pathway of increasing lipolysis in adipose tissue. Specifically, skeletal muscle FFAs uptake increases at the beginning of exercise; however, exercise for more than 15 min increases TG lipolysis in adipose tissue, which surpasses skeletal muscle FFAs uptake due to the membrane diffusion barriers, and finally leads to increased concentrations of plasma FFAs (46, 50). FFAs are incorporated in phospholipids which may increase levels of plasma phospholipid MUFAs (46–49).

The reasons why MVPA increased oleic acid and eicosenoic acid at 15–26 GWs need further investigation. It could be because lipogenesis is very active in the second trimester, and maternal fat mass in adipose tissue reaches the peak at the end of the second trimester (3, 5). Then habitual PA could induce more FFAs generated from adipose tissue lipolysis which are later incorporated in phospholipids (46–49). Finally, significant positive associations of MVPA with plasma phospholipid oleic acid and eicosenoic acid existed exclusively at 15–26 GWs.

As the study measured individual MUFAs as percentages of total FAs, the relative level of one individual MUFA also reflected and accounted for compositions of other individual MUFAs (Supplementary Table 9). The methods using percentages of FAs instead of absolute values have been validated and commonly used in epidemiological studies (51) because absolute values of individual FAs may be masked by individuals’ total lipids levels (52, 53). Meanwhile, studies have suggested that percentages of FAs have more stable and stronger relations with dietary FAs intake and are better predictors for disease (51, 54–56) compared to absolute values of FAs.

### Strengths and limitations

As this is the first study investigating the associations of PA with individual plasma phospholipid MUFAs in pregnant women, our study has several notable strengths. This study measured plasma individual MUFAs at four-time points across pregnancy with concurrent reports of PA which reflected PA before the time of blood samples collection ensuring the temporality of associations. In addition, this study collected rich data allowing us to account for a variety of potential confounders, including dietary intake. Some potential limitations of our study are worthy of discussion. First, like most other observational studies, even though we accounted for many potential confounders, residual confounding may still be present. Second, PA in our study was self-reported. However, the PA questionnaire used in this study has been commonly used in epidemiological studies (57–59) and has been validated by PA objectively measured using accelerometers (ActiGraph) among pregnant women with correlation coefficients for sports/exercise ranging from 0.30 to 0.44 (27). Indeed, self-reported MVPA in our study was consistent with objectively measured MVPA by accelerometers among pregnant women in other studies (60, 61). Meanwhile, self-reported pre-pregnancy weight may be subject to measurement error. However, it was observed that self-reported and directly measured pre-pregnancy weight had very high correlation coefficients among 170 pregnant women from the Project Viva study in the US (*r* = 0.99) (62) and among 2,028 pregnant women from the Southampton Women’s Survey study in the United Kingdom (*r* = 0.977) (63). Third, generalizing our findings to other populations needs to be cautious since our findings are based on a nested case-control study among US pregnant women. However, we did apply sampling weights in the statistical analyses so the results of this study could be representative of the full NICHD Fetal Growth Studies-Singletons cohort (26), which included diverse participants with regard to geographical regions and racial/ethnic groups.

In conclusion, in this prospective and longitudinal study, we found that maternal MVPA was positively associated with individual plasma phospholipid MUFAs (i.e., oleic acid, eicosenoic acid, and palmitoleic acid) during middle-to-late pregnancy even after the adjustment of major confounders, including BMI and dietary factors. These findings suggested that MVPA may be a potentially modifiable factor for plasma individual MUFA levels during pregnancy. Future studies are warranted to confirm these findings.

## Data availability statement

The original contributions presented in this study are included in the article, further inquiries can be directed to the corresponding author.

## Ethics statement

The study was approved by the institutional review boards of NICHD and all participating clinical sites. The IRB approval number is NICHD (09-CH-N152). Before data collection, all participants provided written informed consent.

## Author contributions

TX, LC, and CZ: study design and conceptualization. CZ: funding acquisition, supervision, and data acquisition. SH, YZ, JW, NW, and MT: data management and curation. TX, LC, ZF, XL, and JD: statistical methods and analysis. TX and LC: manuscript drafting. All authors have revised, edited, read, and approved the final manuscript.

## Abbreviations

AHEI: Alternative Health Eating Index
ASA24: automated self-administered 24-h dietary recall
BMI: body mass index
CV: coefficient of variation
ELOVL: elongation of very long chain fatty acids protein
FAs: fatty acids
FFA: free fatty acids
FFQ: food frequency questionnaire
GC-FID: gas chromatography with flame ionization detection
GDM: gestational diabetes mellitus
GWs: gestational weeks
HDL: high-density lipoprotein
IL: interleukin
MUFAs: monounsaturated fatty acids
MVPA: moderate-to-vigorous physical activity
NICHD: National Institute of Child Health and Human Development
PA: physical activity
PPAQ: Pregnancy Physical Activity Questionnaire
PPARγ: peroxisome proliferator-activated receptor gamma
SE: standard error
SFAs: saturated fatty acids
TG: triglyceride
US: United States.
