# Supplementary material for: A longitudinal study on associations of moderate-to-vigorous physical activity with plasma monounsaturated fatty acids in pregnancy
Source: Front Nutr. 2022 Oct 24;9:983418. doi: 10.3389/fnut.2022.983418 (PMC9637551; doi:10.3389/fnut.2022.983418)
Supplement: Supplementary file 1 [file Data_Sheet_1.pdf]

Supplementary material

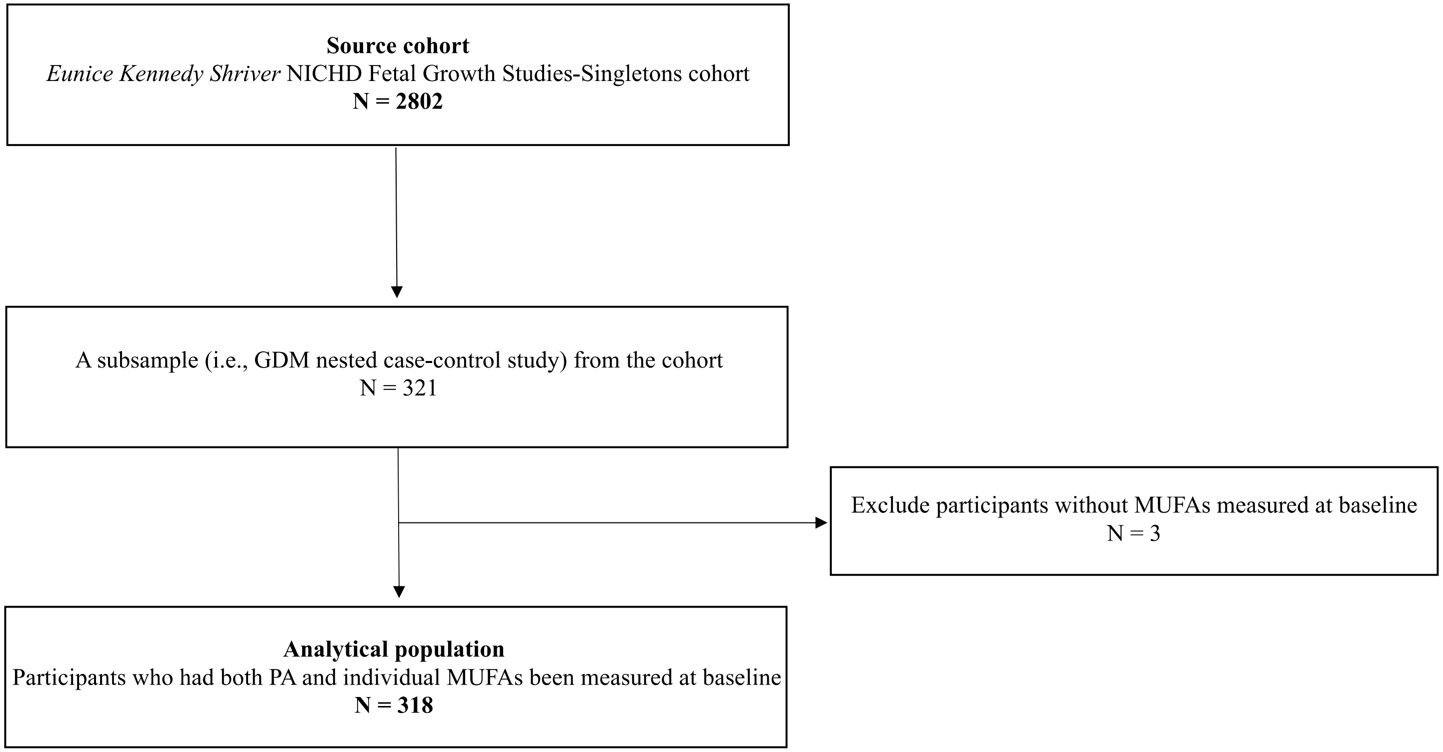

Supplementary Figure 1. Participants flowchart
Abbreviations: GDM, gestational diabetes mellitus; MUFAs, monounsaturated fatty acids; NICHD, National Institute of Child Health and Human Development; PA, physical activity.

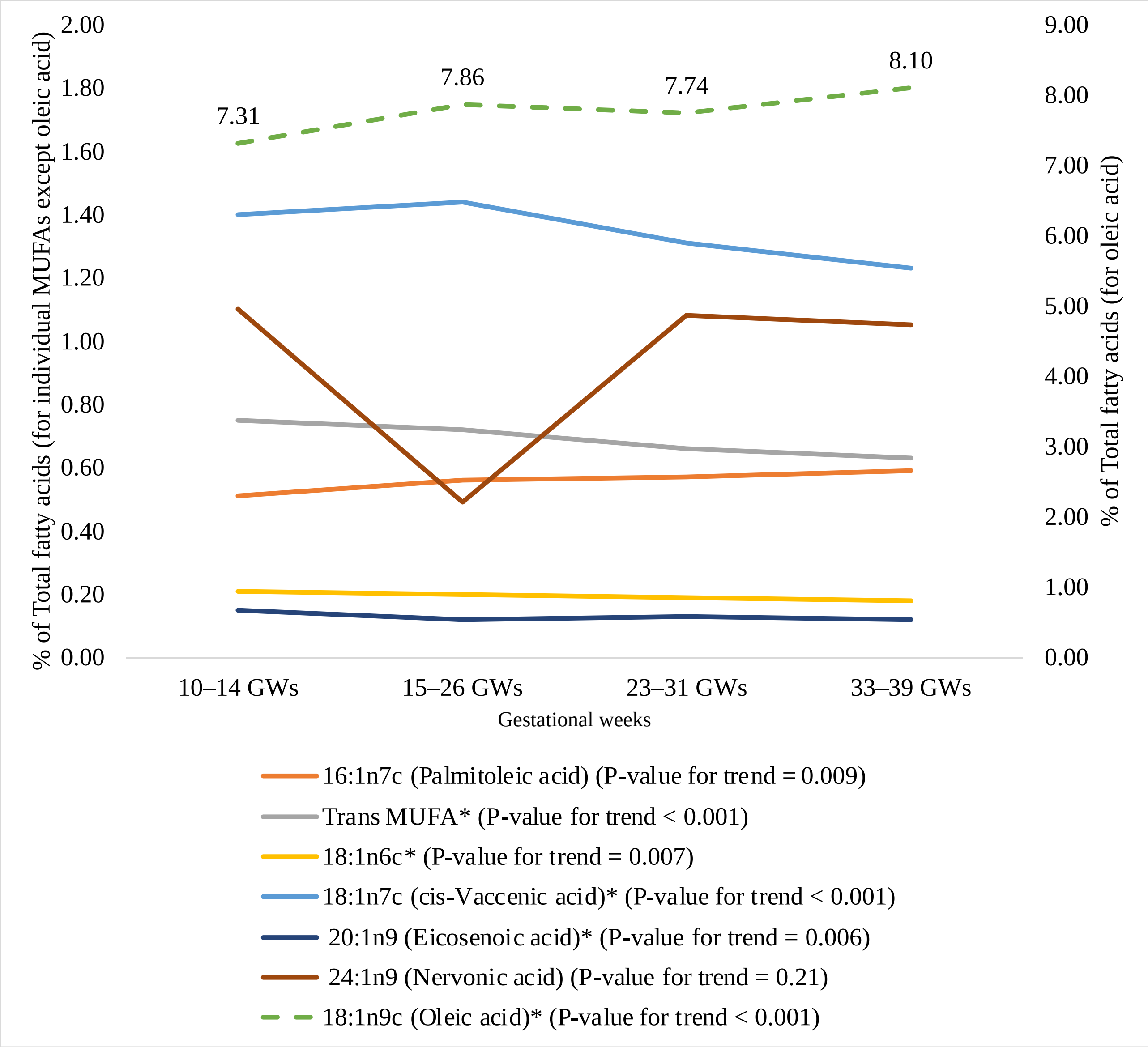

Supplementary Figure 2. Changes of individual plasma phospholipid monounsaturated fatty acids (MUFAs) during pregnancy. Trans MUFA: 18:1n6-9 trans (sum of 18:1n-6 trans, 18:1n-7 trans, 18:1n-8 trans and 18:1n-9 trans). Abbreviations: GWs, gestational weeks; MUFAs, monounsaturated fatty acids.

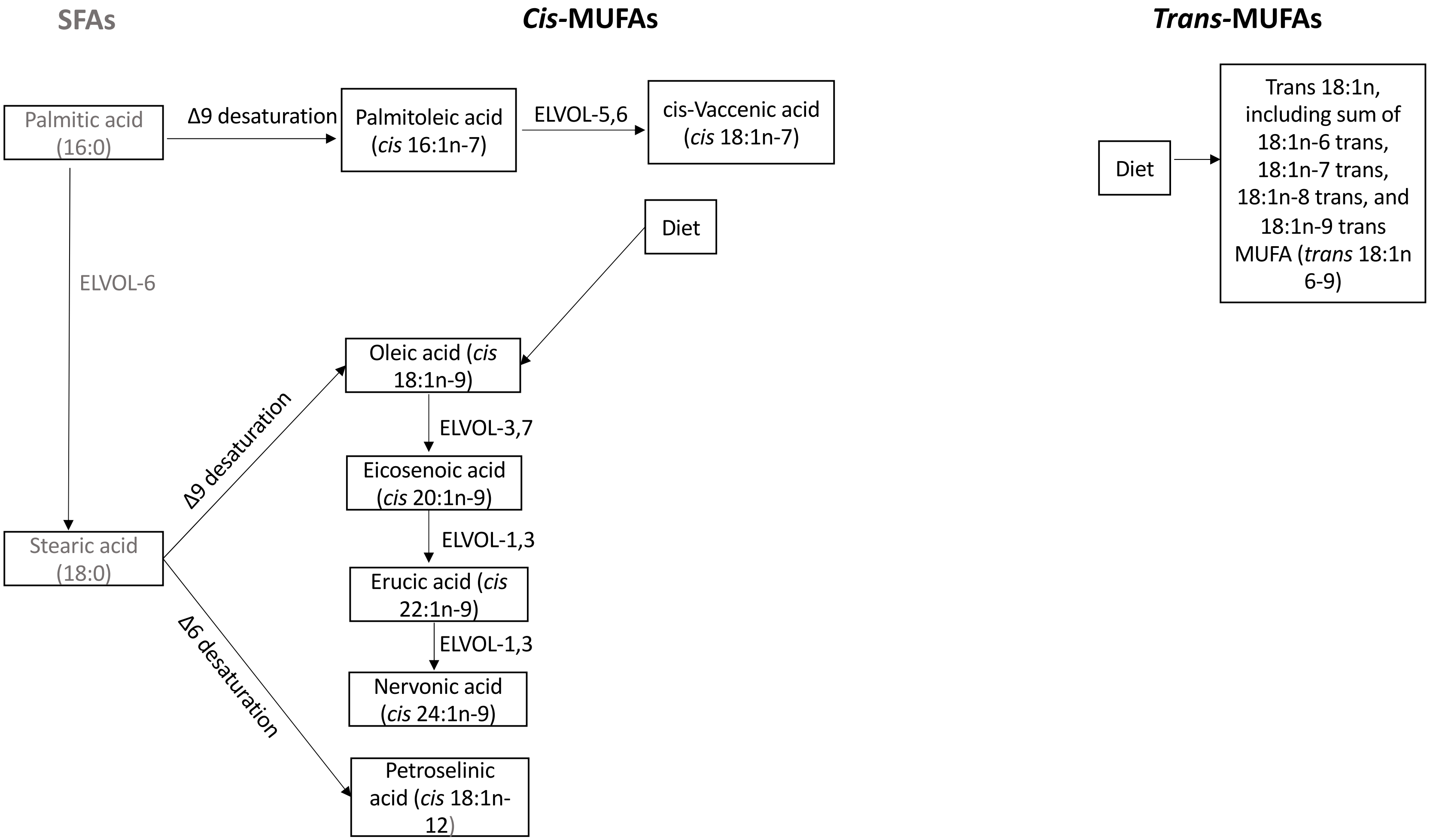

Supplementary Figure 3. Biosynthesis pathways of monounsaturated fatty acids (MUFAs) (1–6)
Cis-MUFAs are from both exogenous (via dietary intake) and endogenous (via de novo lipogenesis, desaturase and elongation) origins. Among which, Oleic acid (*cis* 18:1n-9) accounts for more than 92 % of all cis-MUFAs consumed. Other *cis*-MUFAs are also present in the diet, but only in very small amounts. *Trans*-MUFAs are from exogenous origin.
Abbreviations: ELVOL, elongation of very long chain fatty acids protein; MUFAs, monounsaturated fatty acids; SFAs, saturated fatty acids.

Supplementary Table 1. Distribution of missing variables of interest[1]

	PA			MUFAs[2]			Dietary intakes[3]		
	**N**	**N of missing**	**% of missing**	**N**	**N of missing**	**% of missing**	**N**	**N of missing**	**% of missing**
10–14 gestational weeks	318	0	0.0	318	0	0.0	197	121	38.1
15–26 gestational weeks	316	2	0.6	308	10	3.1	210	108	34.0
23–31 gestational weeks	306	12	3.8	205	6	1.9	146	172	54.1
33–39 gestational weeks	278	40	12.6	187	24	7.5	129	189	59.4

[1]N and % of missing were calculated among 318 pregnant women.
[2]By design, at 10–14 GWs and 15–26 GWs, MUFAs were measured among all GDM cases and controls. At 23–31 GWs and 33–39 GWs, MUFAs were measured among all cases and one of their controls. We used sampling weights in the statistical analyses to account for such biospecimen collection strategy. Thus, at 23–31 GWs and 33–39 GWs, even though the N of MUFAs was 205 and 187, respectively, the actual N of MUFAs was 312 and 294, respectively.
[3]Dietary intakes were assessed during pregnancy at 8–13 GWs, 16–22 GWs, 24–29 GWs, and 34–37 GWs.
Abbreviations: GWs, gestational weeks; MUFAs, monounsaturated fatty acids; PA, physical activity.

Supplementary Table 2. Moderate-to-vigorous physical activity (MVPA) and sedentary behaviors at each study visit[1]

**Time spent on activities**	**10–14 gestational weeks**		**15–26 gestational weeks**		**23–31 gestational weeks**		**33–39 gestational weeks**	
MVPA (hours/week)[2]	2.92	(0.15)	1.98	(0.12)	2.29	(0.13)	1.67	(0.12)
Sedentary behavior (hours/week)[2]	48.70	(1.40)	47.38	(1.46)	43.31	(1.23)	37.34	(1.24)
% of high sedentary behavior[3]	56.34	(185)	54.68	(164)	53.15	(159)	36.53	(116)

[1]Previous year habitual PA was measured at baseline and PA since last visit was measured at following visits. Sampling weights were applied to all analyses to represent the full NICHD Fetal Growth Studies–Singletons population. Women were classified into high sedentary behavior group if they had ≥ 42 hours of sedentary behavior per week.

[2]Data were presented as weighted mean (SE). [3]Data were presented as weighted percentage and actual frequency, % (N).

Abbreviations: MVPA, moderate-to-vigorous physical activity; NICHD, National Institute of Child Health and Human Development; PA, physical activity; SE, standard error.

Supplementary Table 3. Plasma monounsaturated fatty acids (MUFAs) levels at each study visit[1]

	10–14 gestational weeks		15–26 gestational weeks		23–31 gestational weeks		33–39 gestational weeks	
**Total MUFAs**	11.43	(0.06)	11.40	(0.07)	11.68	(0.08)	11.91	(0.08)
16:1n7c (Palmitoleic acid)	0.51	(0.01)	0.56	(0.01)	0.57	(0.02)	0.59	(0.02)
Trans MUFA	0.75	(0.01)	0.72	(0.01)	0.66	(0.02)	0.63	(0.02)
18:1n6c	0.21	(0.004)	0.20	(0.005)	0.19	(0.01)	0.18	(0.01)
18:1n7c (cis-Vaccenic acid)	1.40	(0.01)	1.44	(0.01)	1.31	(0.01)	1.23	(0.01)
18:1n9c (Oleic acid)	7.31	(0.05)	7.86	(0.06)	7.74	(0.07)	8.10	(0.07)
20:1n9 (Eicosenoic acid)	0.15	(0.004)	0.12	(0.004)	0.13	(0.004)	0.12	(0.003)
24:1n9 (Nervonic acid)	1.10	(0.03)	0.49	(0.01)	1.08	(0.03)	1.05	(0.04)

[1]Data were presented as weighted mean (SE). Sampling weights were applied to all analyses to represent the full NICHD Fetal Growth Studies–Singletons population. Individual MUFAs were expressed as % of total fatty acids. Trans MUFA: 18:1n6-9 trans (sum of 18:1n-6 trans, 18:1n-7 trans, 18:1n-8 trans and 18:1n-9 trans).

Abbreviations: MUFAs, monounsaturated fatty acids; NICHD, National Institute of Child Health and Human Development; SE, standard error.

Supplementary Table 4. Associations of moderate-to-vigorous physical activity (MVPA) with plasma monounsaturated fatty acids (MUFAs) using multiple imputation for missing data[1]

	10–14 gestational weeks			15–26 gestational weeks				23–31 gestational weeks				33–39 gestational weeks		
	**$\beta*10^2$**	**($SE*10^2$)**	**P**	**$\beta*10^2$**	**($SE*10^2$)**	**P**		**$\beta*10^2$**	**($SE*10^2$)**	**P**		**$\beta*10^2$**	**($SE*10^2$)**	**P**
**MVPA (hours/week)**														
**Total MUFAs**	5.83	(2.47)	0.02	10.66	(3.16)	0.001	*	8.38	(3.64)	0.02		8.76	(4.68)	0.06
16:1n7c (Palmitoleic acid)	1.00	(0.46)	0.03	0.51	(0.43)	0.24		2.47	(0.66)	<0.001	*	-0.03	(1.00)	0.98
Trans MUFA[2]	0.64	(0.48)	0.18	0.49	(0.70)	0.48		-0.19	(0.66)	0.78		0.20	(0.81)	0.81
18:1n6c	0.30	(0.18)	0.09	0.29	(0.24)	0.22		0.91	(0.34)	0.01		0.32	(0.34)	0.34
18:1n7c (cis-Vaccenic acid)	-0.29	(0.47)	0.53	0.55	(0.55)	0.32		-0.56	(0.62)	0.36		0.43	(0.59)	0.47
18:1n9c (Oleic acid)	4.02	(2.00)	0.04	8.68	(2.62)	0.001	*	4.88	(2.89)	0.09		7.80	(3.86)	0.05
20:1n9 (Eicosenoic acid)	-0.16	(0.15)	0.29	0.55	(0.20)	0.01	*	0.36	(0.17)	0.03		0.27	(0.19)	0.16
24:1n9 (Nervonic acid)	0.37	(1.17)	0.75	-0.44	(0.51)	0.39		0.52	(1.44)	0.72		0.64	(1.97)	0.75

[1]Multivariable models adjusted for age (years), race, education, marriage status, nulliparous, pre-pregnancy BMI (kg/m^2), AHEI score, and sedentary behavior (hour/week)**. Multiple imputation was used for the missing data.** Sampling weights were applied to all analyses to represent the full NICHD Fetal Growth Studies–Singletons population. *P-value is significant after Bonferroni correction. Previous year habitual PA was measured at baseline and PA since last visit was measured at following visits. Individual MUFAs were expressed as % of total fatty acids.

[2]Trans MUFA: 18:1n6-9 trans (sum of 18:1n-6 trans, 18:1n-7 trans, 18:1n-8 trans and 18:1n-9 trans).

Abbreviations: AHEI, Alternative Health Eating Index; BMI, body mass index; MUFAs, monounsaturated fatty acids; MVPA, moderate-to-vigorous physical activity; NICHD, National Institute of Child Health and Human Development; PA, physical activity; SE, standard error.

Supplementary Table 5. Associations of moderate-to-vigorous physical activity (MVPA) with plasma monounsaturated fatty acids (MUFAs) further controlling for diet source of fatty acids[1]

	**10–14 gestational weeks**				**15–26 gestational weeks**				**23–31 gestational weeks**				**33–39 gestational weeks**		
	**$\beta*10^2$**	**($SE*10^2$)**	**P**		**$\beta*10^2$**	**($SE*10^2$)**	**P**		**$\beta*10^2$**	**($SE*10^2$)**	**P**		**$\beta*10^2$**	**($SE*10^2$)**	**P**
**MVPA (hours/week)**															
**Total MUFAs**	5.03	(2.35)	0.03		10.43	(3.19)	0.001	*	6.54	(3.58)	0.07		6.40	(4.33)	0.14
16:1n7c (Palmitoleic acid)	0.72	(0.44)	0.10		0.52	(0.43)	0.22		2.25	(0.65)	0.001	*	-0.04	(0.84)	0.96
Trans MUFA[2]	0.56	(0.46)	0.23		0.40	(0.69)	0.56		-0.42	(0.65)	0.52		-0.26	(0.77)	0.73
18:1n6c	0.26	(0.17)	0.13		0.30	(0.23)	0.20		0.69	(0.34)	0.04		0.19	(0.31)	0.54
18:1n7c (cis-Vaccenic acid)	-0.30	(0.44)	0.50		0.63	(0.55)	0.26		-0.45	(0.61)	0.46		0.34	(0.59)	0.57
18:1n9c (Oleic acid)	3.49	(1.91)	0.07		8.50	(2.64)	0.001	*	3.13	(2.80)	0.26		5.26	(3.49)	0.13
20:1n9 (Eicosenoic acid)	-0.15	(0.15)	0.30		0.56	(0.20)	0.01	*	0.29	(0.16)	0.07		0.19	(0.19)	0.32
24:1n9 (Nervonic acid)	0.43	(1.13)	0.70		-0.44	(0.52)	0.40		1.18	(1.41)	0.40		1.02	(1.88)	0.59

[1]Multivariable models adjusted for age (years), race, education, marriage status, nulliparous, pre-pregnancy BMI (kg/m^2), AHEI score, sedentary behavior (hour/week), and **diet source of fatty acids.** AHEI score and diet source fatty acids used mean imputation for missing values. Sampling weights were applied to all analyses to represent the full NICHD Fetal Growth Studies–Singletons population. *P-value is significant after Bonferroni correction. Previous year habitual PA was measured at baseline and PA since last visit was measured at following visits. Individual MUFAs were expressed as % of total fatty acids.

[2]Trans MUFA: 18:1n6-9 trans (sum of 18:1n-6 trans, 18:1n-7 trans, 18:1n-8 trans and 18:1n-9 trans).

Abbreviations: AHEI, Alternative Health Eating Index; BMI, body mass index; MUFAs, monounsaturated fatty acids; MVPA, moderate-to-vigorous physical activity; NICHD, National Institute of Child Health and Human Development; PA, physical activity; SE, standard error.

Supplementary Table 6. Associations of moderate-to-vigorous physical activity (MVPA) with plasma monounsaturated fatty acids (MUFAs) further controlling for total energy intake[1]

	10–14 gestational weeks			15–26 gestational weeks			23–31 gestational weeks			33–39 gestational weeks		
	**β*10^2**	**(SE*10^2)**	**P**	**β*10^2**	**(SE*10^2)**	**P**	**β*10^2**	**(SE*10^2)**	**P**	**β*10^2**	**(SE*10^2)**	**P**
**MVPA (hours/week)**												
**Total MUFAs**	5.03	(2.35)	0.03	10.41	(3.19)	0.001 *	6.00	(3.56)	0.09	6.51	(4.37)	0.14
16:1n7c (Palmitoleic acid)	0.73	(0.44)	0.10	0.50	(0.43)	0.25	2.25	(0.65)	0.001 *	-0.26	(0.87)	0.76
Trans MUFA[2]	0.56	(0.46)	0.23	0.37	(0.69)	0.59	-0.30	(0.66)	0.64	-0.25	(0.77)	0.75
18:1n6c	0.27	(0.17)	0.11	0.26	(0.24)	0.27	0.74	(0.34)	0.03	0.14	(0.31)	0.66
18:1n7c (cis-Vaccenic acid)	-0.31	(0.44)	0.48	0.63	(0.55)	0.26	-0.66	(0.60)	0.27	0.34	(0.60)	0.57
18:1n9c (Oleic acid)	3.49	(1.90)	0.07	8.53	(2.63)	0.001 *	2.50	(2.77)	0.37	5.17	(3.49)	0.14
20:1n9 (Eicosenoic acid)	-0.15	(0.15)	0.30	0.55	(0.20)	0.01 *	0.27	(0.16)	0.10	0.21	(0.19)	0.26
24:1n9 (Nervonic acid)	0.44	(1.13)	0.70	-0.44	(0.52)	0.40	1.21	(1.41)	0.39	1.15	(1.87)	0.54

[1]Multivariable models adjusted for age (years), race, education, marriage status, nulliparous, pre-pregnancy BMI (kg/m^2), AHEI score, sedentary behavior (hour/week), and **total energy intake.** AHEI score and total energy intake used mean imputation for missing values. Sampling weights were applied to all analyses to represent the full NICHD Fetal Growth Studies–Singletons population. *P-value is significant after Bonferroni correction. Previous year habitual PA was measured at baseline and PA since last visit was measured at following visits. Individual MUFAs were expressed as % of total fatty acids.

[2]Trans MUFA: 18:1n6-9 trans (sum of 18:1n-6 trans, 18:1n-7 trans, 18:1n-8 trans and 18:1n-9 trans).

Abbreviations: AHEI, Alternative Health Eating Index; BMI, body mass index; MUFAs, monounsaturated fatty acids; MVPA, moderate-to-vigorous physical activity; NICHD, National Institute of Child Health and Human Development; PA, physical activity; SE, standard error.

Supplementary Table 7. Associations of moderate-to-vigorous physical activity (MVPA) with plasma monounsaturated fatty acids (MUFAs) among pregnant women without GDM[1]

	10–14 gestational weeks			15–26 gestational weeks			23–31 gestational weeks			33–39 gestational weeks		
	**β*10²**	**(SE*10²)**	**P**	**β*10²**	**(SE*10²)**	**P**	**β*10²**	**(SE*10²)**	**P**	**β*10²**	**(SE*10²)**	**P**
**MVPA (hours/week)**												
**Total MUFAs**	4.12	(2.89)	0.15	7.85	(3.94)	0.05	6.99	(5.48)	0.20	3.40	(6.94)	0.62
16:1n7c (Palmitoleic acid)	0.52	(0.50)	0.30	0.35	(0.54)	0.52	1.63	(0.99)	0.10	-0.40	(1.36)	0.77
Trans MUFA[2]	0.47	(0.60)	0.44	1.13	(0.78)	0.14	-0.07	(0.97)	0.94	0.59	(1.18)	0.62
18:1n6c	0.24	(0.22)	0.26	0.57	(0.28)	0.05	0.87	(0.52)	0.09	0.40	(0.48)	0.41
18:1n7c (cis-Vaccenic acid)	-0.11	(0.56)	0.84	0.32	(0.67)	0.63	-0.17	(0.87)	0.84	0.03	(0.96)	0.98
18:1n9c (Oleic acid)	2.31	(2.33)	0.32	5.55	(3.14)	0.08	4.56	(4.23)	0.28	2.17	(5.35)	0.69
20:1n9 (Eicosenoic acid)	-0.09	(0.19)	0.63	0.45	(0.23)	0.05	0.37	(0.23)	0.11	-0.05	(0.30)	0.87
24:1n9 (Nervonic acid)	0.78	(1.26)	0.54	-0.52	(0.64)	0.43	-0.19	(1.89)	0.92	0.66	(2.80)	0.81

[1]Multivariable models adjusted for age (years), race, education, marriage status, nulliparous, pre-pregnancy BMI (kg/m²), AHEI score and sedentary behavior (hour/week). AHEI score used mean imputation for missing values. **Sampling weights were not applied**. *P-value is significant after Bonferroni correction. Previous year habitual PA was measured at baseline and PA since last visit was measured at following visits. Individual MUFAs were expressed as % of total fatty acids.
[2]Trans MUFA: 18:1n6-9 trans (sum of 18:1n-6 trans, 18:1n-7 trans, 18:1n-8 trans and 18:1n-9 trans).
Abbreviations: AHEI, Alternative Health Eating Index; BMI, body mass index; GDM, gestational diabetes mellitus; MUFAs, monounsaturated fatty acids; MVPA, moderate-to-vigorous physical activity; PA, physical activity; SE, standard error.

Supplementary Table 8. Associations of moderate-to-vigorous physical activity (MVPA) with fatty acid ratios[1]

	10–14 gestational weeks			15–26 gestational weeks			23–31 gestational weeks				33–39 gestational weeks		
	**β*10²**	**(SE*10²)**	**P**	**β*10²**	**(SE*10²)**	**P**	**β*10²**	**(SE*10²)**	**P**		**β*10²**	**(SE*10²)**	**P**
**MVPA (hours/week)**													
Palmitoleic acid/Palmitic acid (Δ9-desaturase)	0.02	(0.01)	0.14	0.02	(0.01)	0.10	0.07	(0.02)	<0.001	*	-0.01	(0.03)	0.83
cis-Vaccenic acid/Palmitoleic acid (ELOVL-5, 6)	-2.96	(2.63)	0.26	-1.18	(2.15)	0.58	-9.90	(3.08)	0.001	*	0.97	(4.11)	0.81

[1]Multivariable models adjusted for age (years), race, education, marriage status, nulliparous, pre-pregnancy BMI (kg/m²), AHEI score and sedentary behavior (hour/week). AHEI score used mean imputation for missing values. Sampling weights were applied to all analyses to represent the full NICHD Fetal Growth Studies–Singletons population. *P-value is significant after Bonferroni correction. Previous year habitual PA was measured at baseline and PA since last visit was measured at following visits. Individual FAs were expressed as % of total fatty acids.
Abbreviations: AHEI, Alternative Health Eating Index; BMI, body mass index; ELOVL-5, 6, elongation of very long chain fatty acids protein-5, 6; MVPA, moderate-to-vigorous physical activity; NICHD, National Institute of Child Health and Human Development; PA, physical activity; SE, standard error.

Supplementary Table 9. Correlations between the individual plasma monounsaturated fatty acids (MUFAs)

10–14 gestational weeks							
	16:1n7c (Palmitoleic acid)	Trans MUFA[1]	18:1n6c	18:1n7c (cis-Vaccenic acid)	18:1n9c (Oleic acid)	20:1n9 (Eicosenoic acid)	24:1n9 (Nervonic acid)
16:1n7c (Palmitoleic acid)	1.00	-0.06	0.18	-0.07	0.26	0.04	0.22
Trans MUFA[1]	-0.06	1.00	0.74	-0.09	-0.08	0.14	0.11
18:1n6c	0.18	0.74	1.00	-0.11	-0.02	0.27	0.20
18:1n7c (cis-Vaccenic acid)	-0.07	-0.09	-0.11	1.00	0.15	0.15	0.09
18:1n9c (Oleic acid)	0.26	-0.08	-0.02	0.15	1.00	0.07	-0.27
20:1n9 (Eicosenoic acid)	0.04	0.14	0.27	0.15	0.07	1.00	0.18
24:1n9 (Nervonic acid)	0.22	0.11	0.20	0.09	-0.27	0.18	1.00
**15–26 gestational weeks**							
	16:1n7c (Palmitoleic acid)	Trans MUFA[1]	18:1n6c	18:1n7c (cis-Vaccenic acid)	18:1n9c (Oleic acid)	20:1n9 (Eicosenoic acid)	24:1n9 (Nervonic acid)
16:1n7c (Palmitoleic acid)	1.00	-0.19	-0.06	0.09	0.53	0.02	-0.18
Trans MUFA[1]	-0.19	1.00	0.83	0.03	-0.08	0.12	-0.12
18:1n6c	-0.06	0.83	1.00	0.04	0.09	0.24	-0.18
18:1n7c (cis-Vaccenic acid)	0.09	0.03	0.04	1.00	0.26	0.29	0.03
18:1n9c (Oleic acid)	0.53	-0.08	0.09	0.26	1.00	0.30	-0.09
20:1n9 (Eicosenoic acid)	0.02	0.12	0.24	0.29	0.30	1.00	-0.24
24:1n9 (Nervonic acid)	-0.18	-0.12	-0.18	0.03	-0.09	-0.24	1.00
**23–31 gestational weeks**							
	16:1n7c (Palmitoleic acid)	Trans MUFA[1]	18:1n6c	18:1n7c (cis-Vaccenic acid)	18:1n9c (Oleic acid)	20:1n9 (Eicosenoic acid)	24:1n9 (Nervonic acid)
16:1n7c (Palmitoleic acid)	1.00	-0.31	0.00	-0.03	0.53	0.10	-0.13
Trans MUFA[1]	-0.31	1.00	0.76	-0.13	-0.07	0.21	0.07

18:1n6c	0.00	0.76	1.00	-0.06	0.12	0.22	0.01
18:1n7c (cis-Vaccenic acid)	-0.03	-0.13	-0.06	1.00	0.15	0.29	-0.08
18:1n9c (Oleic acid)	0.53	-0.07	0.12	0.15	1.00	0.16	-0.31
20:1n9 (Eicosenoic acid)	0.10	0.21	0.22	0.29	0.16	1.00	0.06
24:1n9 (Nervonic acid)	-0.13	0.07	0.01	-0.08	-0.31	0.06	1.00

33–39 gestational weeks

	16:1n7c (Palmitoleic acid)	Trans MUFA[1]	18:1n6c	18:1n7c (cis-Vaccenic acid)	18:1n9c (Oleic acid)	20:1n9 (Eicosenoic acid)	24:1n9 (Nervonic acid)
16:1n7c (Palmitoleic acid)	1.00	-0.15	0.11	-0.24	0.41	0.11	-0.05
Trans MUFA[1]	-0.15	1.00	0.82	-0.14	0.04	0.18	0.15
18:1n6c	0.11	0.82	1.00	-0.10	0.12	0.27	0.13
18:1n7c (cis-Vaccenic acid)	-0.24	-0.14	-0.10	1.00	0.00	0.17	-0.01
18:1n9c (Oleic acid)	0.41	0.04	0.12	0.00	1.00	0.21	-0.32
20:1n9 (Eicosenoic acid)	0.11	0.18	0.27	0.17	0.21	1.00	0.19
24:1n9 (Nervonic acid)	-0.05	0.15	0.13	-0.01	-0.32	0.19	1.00

Sampling weights were applied. Individual MUFA was measured as % of total fatty acids.
[1] Trans MUFA: 18:1n6-9 trans (sum of 18:1n-6 trans, 18:1n-7 trans, 18:1n-8 trans and 18:1n-9 trans).
Abbreviations: MUFA, monounsaturated fatty acids

References

1. Tvrzicka E, Kremmyda LS, Stankova B, Zak A. FATTY ACIDS AS BIOCOMPOUNDS: THEIR ROLE IN HUMAN METABOLISM, HEALTH AND DISEASE - A REVIEW. PART 1: CLASSIFICATION, DIETARY SOURCES AND BIOLOGICAL FUNCTIONS. Biomed Pap. 2011 Jun 1;155(2):117–30.

2. Raatz SK, Bibus D, Thomas W, Kris-Etherton P. Total Fat Intake Modifies Plasma Fatty Acid Composition in Humans. J Nutr. 2001 Feb 1;131(2):231–4.

3. Miyazaki M, Ntambi JM. Fatty acid desaturation and chain elongation in mammals. In: Biochemistry of Lipids, Lipoproteins and Membranes [Internet]. Elsevier; 2008 [cited 2021 Nov 11]. p. 191–211. Available from: https://linkinghub.elsevier.com/retrieve/pii/B978044453219050009X

4. Sampath H, Ntambi JM. Role of stearoyl-CoA desaturase in human metabolic disease. Future Lipidology. 2008 Apr;3(2):163–73.

5. Kihara A. Synthesis and degradation pathways, functions, and pathology of ceramides and epidermal acylceramides. Progress in Lipid Research. 2016 Jul;63:50–69.

6. Green CD, Ozguden-Akkoc CG, Wang Y, Jump DB, Olson LK. Role of fatty acid elongases in determination of de novo synthesized monounsaturated fatty acid species. J Lipid Res. 2010 Jul;51(7):1871–7.
